# Microbiota-mediated mechanisms of natural products in atherosclerosis: focus on metabolic and inflammatory pathways

**DOI:** 10.3389/fendo.2026.1818349

**Published:** 2026-06-26

**Authors:** Qi Shen, Ce Bian, Mengjun Zhou, Xingkun Shen, Xiaodong Jin, Bo Li

**Affiliations:** 1 School of Clinical Medicine, Shandong Second Medical University, Weifang, Shandong, China; 2 Department of Cardiology, Binzhou Medical University, Yantai, Shandong, China; 3 School of Clinical Medicine, Jining Medical University, Jining, Shandong, China; 4 Department of Geriatrics, Zibo Central Hospital, Zibo, Shandong, China; 5 Department of Cardiology, Zibo Central Hospital, Zibo, Shandong, China

**Keywords:** atherosclerosis, bile acid metabolism, gut microbiota, natural products, SCFAs, TMAO

## Abstract

**Background:**

Atherosclerosis (AS) is a chronic inflammatory vascular disease characterized by lipid accumulation, endothelial dysfunction, immune dysregulation, and plaque formation. Beyond conventional lipid-related mechanisms, gut microbiota dysbiosis and microbiota-derived metabolites have emerged as important regulators of atherogenesis. Natural products, including polyphenols, flavonoids, alkaloids, fatty acids, polysaccharides, saponins, and terpenoids, may modulate AS by reshaping gut microbial ecology and metabolic outputs.

**Methods:**

This narrative review qualitatively synthesized English-language studies published from 2016 to 2026, with emphasis on recent preclinical and emerging clinical evidence. Literature was retrieved from PubMed and Google Scholar using terms related to natural products, gut microbiota, and atherosclerosis. Evidence was integrated across natural product categories, microbial metabolites, host signaling pathways, preclinical models, clinical observations, and translational limitations.

**Results:**

Natural products consistently acted on convergent microbiota-dependent pathways rather than isolated mechanisms. They reduced trimethylamine/trimethylamine N-oxide production, promoted short-chain fatty acid generation, remodeled bile acid metabolism, and modulated microbial tryptophan-derived metabolites. These metabolic changes were associated with improved intestinal barrier integrity, suppression of TLR4/NF-κB and NLRP3-mediated inflammation, immune rebalancing, reduced oxidative stress, enhanced cholesterol efflux, and attenuation of plaque-related phenotypes. Polyphenols and berberine showed relatively stronger mechanistic support, whereas polysaccharides, saponins, terpenoids, and complex formulas remain mainly exploratory. Most evidence derives from animal and *in vitro* studies, while clinical studies remain limited by small samples, short follow-up, heterogeneous interventions, surrogate endpoints, and insufficient causal validation.

**Conclusions:**

Natural products provide an integrated framework for targeting the gut microbiota–metabolite–vascular pathology axis in AS. Although current evidence supports their biological plausibility and adjunctive therapeutic potential, standardized preparations, causal microbiome validation, multi-omics-based biomarkers, and well-designed clinical trials with vascular or cardiovascular endpoints are required before clinical translation.

## 1 Introduction

Atherosclerosis (AS) is the main pathological basis of major cardiovascular diseases and one of the leading causes of morbidity and mortality worldwide (1). Its core characteristics are lipid deposition in the arterial intima, inflammatory cell infiltration, endothelial dysfunction and vascular remodeling which eventually forms plaques and leads to luminal narrowing (1). Although lipid-lowering therapies especially statins have significantly reduced the incidence of cardiovascular events, many high-risk patients still have significant residual risk (2). This phenomenon suggests that in addition to blood lipid disorders, other mechanisms also play an important role in the progression of atherosclerosis and new prevention and control strategies are urgently needed (3).

Among these emerging mechanisms, the gut microbiota has attracted increasing attention as a key regulatory factor for the development of AS. Gut microbial dysbiosis can affect the host’s lipid metabolism, inflammatory response, immune homeostasis and endothelial function, thereby promoting the formation of AS (4–6). The above effects are partly mediated by microbiota-derived metabolites, including trimethylamine N-oxide (TMAO), short-chain fatty acids (SCFAs) and bile acid-related metabolites, which can have harmful or protective effects on blood vessel health (7–9). The increase in TMAO levels in circulation is closely related to increased cholesterol accumulation, endothelial dysfunction and vascular inflammation, supporting its pro-atherogenic role (9–11). On the contrary, acetic acid, propionic acid, butyric acid and other SCFAs can improve intestinal barrier integrity, regulate the immune response and maintain metabolic homeostasis, and exert a potential anti-atherosclerotic effect (12, 13). In summary, gut microbiota and their metabolites are highly potential targets for the prevention and treatment of AS (14).

Natural products have become ideal candidates for targeted gut microbiota intervention because of their multi-component, multi-target properties and relatively favorable safety profiles (15, 16). More and more evidence shows that may attenuate AS-related phenotypes by reshaping the structure of gut microbiota, regulating microbial metabolism, reducing the generation of atherosclerotic metabolites, and restoring the balance of host metabolism and inflammation (17). However, the existing research is relatively fragmented in terms of natural product categories, microbial targets, metabolites and host signaling pathways, especially lacking an integrative narrative synthesis of how natural products regulate gut microbiota and their functional outputs to affect the progression of AS. The lack of integrated research makes it difficult to build a coherent mechanism framework and assess the translational potential of such interventions.

Therefore, this article reviews the research status of natural products as gut microbiota regulators in AS, explains the main categories of natural products, their regulatory effects on gut microbiota and metabolites, and the downstream mechanisms of these changes related to lipid metabolism, inflammation, immune response, endothelial function and plaque progression. At the same time, the current limitations of this field are pointed out, and the future direction of the clinical translation of natural product therapy targeting gut microbiota in AS is discussed.

## 2 Methods

The present work is a narrative review based on a qualitative synthesis of the literature. Literature review was carried out using electronic databases: PubMed and Google Scholar. The following keywords were used: “natural products,” “gut microbiota,” “atherosclerosis.” Inclusion criteria included: only original research papers, meta-analyses, and review articles published from 2016 to 2026, with a particular emphasis on the last five years, and original publications in English. Exclusion criteria included: studies published before 2016, studies in languages other than English, and those outside the scope of the relationship between natural products, gut microbiota, and atherosclerosis. The focus was on the interactions between natural products and gut microbiota in the modulation of atherosclerosis, and less on unrelated aspects such as general effects of natural products on other cardiovascular diseases or microbiota’s role in diseases unrelated to atherosclerosis. The review aimed to synthesize the most recent findings and highlight novel perspectives within this specific intersection.

## 3 Classification of natural products and their effects on gut microbiota

Natural products contain biologically active molecules with diverse structures, most of which have been reported to have the potential to prevent or alleviate atherosclerosis (18, 19). Despite the wide variety, existing studies show that their gut microbiota related effects against atherosclerosis are not through completely independent pathways, but concentrated in a few common mechanisms (20). Among the different categories of natural products, the most commonly involved gut-microbiota dependent processes include inhibiting TMA/TMAO production, restoring SCFA production, regulating bile acid metabolism, improving intestinal barrier integrity, and reducing chronic inflammation (21). Therefore, the significance of natural products in atherosclerosis lies more in their common ability to reshape intestinal microecology and its metabolic output than in category specificity (22).

At the same time, the evidence strength of different types of compounds is uneven. Most mechanism studies are based on animal models or *in vitro* systems. Human evidence is relatively limited and metabolic or inflammatory surrogate endpoints are often predominant rather than direct plaque related outcomes. In addition, for many compounds, it is difficult to distinguish whether the effect is mediated by gut microbiota remodeling or induced by direct actions on host lipid metabolism, oxidative stress or inflammatory signals. Therefore, the discussion in this article not only focuses on the main categories of natural products but also emphasizes shared mechanisms, evidence reliability and key limitations that require attention when interpreting existing literature (23) (**Table 1**).

**Table 1:** Product-centered summary of natural products targeting the gut microbiota–atherosclerosis axis: major mechanisms, evidence status, and translational limitations.

Natural product/representative intervention	Product class	Main microbiota-related mechanism(s)	Main AS-related effect(s)	Evidence level	Translational status	Key limitation(s)
Resveratrol	Polyphenol	Suppression of TMA/TMAO pathway; bile acid remodeling; enrichment of *Lactobacillus/Bifidobacterium*	Reduced inflammatory burden; improved cholesterol handling; attenuation of plaque-related phenotypes	Animal + limited human-related mechanistic evidence	Early translational signal	Human AS-specific evidence remains limited; multiple host-directed effects may coexist
Quercetin / anthocyanins / tea polyphenols	Flavonoids / polyphenols	Enrichment of beneficial commensals; increased SCFA production; reduction of pro-inflammatory taxa	Improved lipid metabolism; reduced inflammation; microbiota normalization	Mostly animal / preclinical	Mechanistically promising	Considerable heterogeneity across compounds; microbiota-specific causality often unproven
Berberine	Alkaloid	Inhibition of microbial TMA production; suppression of TMA-producing taxa/genes; enrichment of SCFA-associated bacteria	Lower circulating TMAO; reduced lesion burden; improved lipid and inflammatory indices	Animal + preliminary clinical observations	One of the strongest translational candidates	Evidence within the alkaloid class is heavily dominated by berberine; direct host pharmacology also contributes
Palmatine	Alkaloid	Remodeling of microbial composition; reduction of unfavorable taxa; altered microbial metabolite profile	Delayed plaque progression; reduced proatherogenic metabolites	Animal only	Preclinical	Limited number of studies; generalizability uncertain
EPA / DHA / fish oil	Natural fatty acids	Partial correction of dysbiosis; barrier support; reduced secondary bile acid burden	Lower systemic inflammation; improved lipid profile; reduced plaque area in models	Animal + broader cardiovascular human literature	Mechanistically relevant but microbiota-specific role less defined	Strong direct host effects make microbiota-mediated contribution difficult to isolate
Dendrobium huoshanense polysaccharides	Polysaccharides	Fermentation-driven microbiota remodeling; bile acid-related metabolic shifts	Reduced oxidative stress; improved microbial profile; anti-AS effects in models	Animal only	Exploratory	Compound category remains heterogeneous; human evidence lacking
Ginsenoside Rb1 / ginseng-derived saponins	Saponins	Microbial biotransformation to active metabolites; microbiota-dependent anti-inflammatory effects	Improved lipid/inflammatory phenotype; possible immune modulation	Mostly animal / mechanistic	Exploratory	Activity depends strongly on microbial conversion; evidence remains fragmented
Tanshinones	Terpenoid / terpenoid quinones	Microbiota modulation; inflammatory pathway suppression	Improvement of AS-related phenotypes in models	Animal only	Exploratory	Limited class-level evidence; mechanisms not consistently microbiota-specific
Naoxintong (NXT)	Multi-component traditional formula	Increased SCFA-associated taxa; altered fecal SCFAs; broader microbiota remodeling	Improved lipid/inflammatory surrogate markers; lesion-related benefit in models	Animal + pilot clinical signal	Clinically preliminary	Complex formulation complicates attribution of active components and mechanisms
Liuwei Dihuang Wan (LWDH)	Multi-component traditional formula	Reduction of TMA-producing taxa; bile acid remodeling; lowered TMAO	Slowed AS progression in models; improved metabolic/inflammatory profile	Animal only	Mechanistically suggestive	Formula complexity and limited causal validation restrict interpretation
Buyang Huanwu Decoction (BYHWD)	Multi-component traditional formula	Microbiota remodeling; reduced TMAO; indirect suppression of gut-derived inflammation	Reduced plaque inflammation and vulnerability-related markers	Animal only	Preclinical	Multi-target effects make microbiota dependence difficult to isolate
Peanut skin polyphenols / red wine polyphenols	Polyphenol-rich extracts	Promotion of butyrate-producing communities; SCFA restoration; inflammatory restraint	Reduced cytokine burden; improved AS-related phenotype in models	Mostly animal	Mechanistically promising	Extract composition varies; taxonomic findings may differ across models

### 3.1 Polyphenols and flavonoids

Polyphenols are one of the most widely studied natural products in the field of gut microbiota and atherosclerosis including phenolic acids, coumarins, lignans, tannins, flavonoids and other subclasses. The key feature of most polyphenols is that they are small and poorly absorbed in the intestine mainly because of their hydrophilicity and complex chemical structure. After a large number of components reach the colon, they are converted into smaller phenolic metabolites through intestinal microorganisms and their bioavailability and biological activity are often higher than those of parent compounds (24). This characteristic makes gut microbiota the core factor that determines the biological activity of polyphenols (25). On the one hand, intestinal microorganisms metabolize polyphenols through deglycosylation, reduction, ring opening and other reactions to produce metabolites that can regulate host oxidation, inflammation and metabolic pathways (26). On the other hand, polyphenols can selectively promote the increase of beneficial commensals and inhibit the growth of bacteria related to inflammation or metabolic disorders to reshape the intestinal microecology (27). These effects are related to changes in bacterial energy metabolism, adhesion and quorum sensing, which ultimately promote the homeostasis of gut microbiota.

Flavonoids are the most deeply studied subclass of polyphenols (28). Representative compounds include quercetin, anthocyanins, hawthorn flavonoids and isoflavones, which are widely found in fruits and vegetables, tea and medicinal plants (29). Similar to the characteristics of polyphenols, flavonoids often reach the colon in the form of glycosylation or polymerization, and rely heavily on the transformation of microorganisms to exert their complete biological effects (26). Their metabolites (multiple phenolic acids) have the effects of anti-oxidation, reducing the release of inflammatory mediators, and improving lipid metabolism. Experimental research results also further show that flavonoids and their metabolites can enrich beneficial bacteria such as *Lactobacillus* and *Bifidobacterium*, inhibit potentially harmful bacteria, and restore the balance of gut microbiota.

Importantly, although a large number of polyphenols and flavonoids are currently being studied, the downstream mechanisms reported for these compounds are highly overlapping. Tea polyphenols can increase the abundance of beneficial bacteria such as *Bifidobacterium* and promote the generation of SCFAs (28, 30). Red wine polyphenols are associated with increased abundance of *Akkermansia* and reduced abundance of pro-inflammatory bacteria (31, 32). Resveratrol, chlorogenic acid, tannic acid, anthocyanins and quercetin have been widely reported, mainly in experimental or preclinical settings, to modulate gut microbiota and improve atherosclerosis-related metabolic or inflammatory phenotypes (33, 34). Some studies have shown that the related effects of such compounds are accompanied by the improvement of blood lipids, the reduction of systemic inflammation or the reduction of plasma TMAO levels. In summary, these findings support the existence of a “polyphenol-microbial metabolism-host response” axis in atherosclerosis (32, 35). However, a large number of studies on polyphenols and flavonoids also highlight the limitations of this field. Although such compounds are one of the most well-evidenced categories of natural products, most relevant studies are still preclinical studies and rely on parallel associations between changes in gut microbiota composition, metabolic changes and atherosclerotic phenotypes. In addition, the mechanisms of different compounds are highly overlapping, and it is difficult to determine whether the benefits mainly come from parent compounds, microbiota-derived metabolites, overall dietary effects or direct host targeting. Therefore, the prospect of polyphenols and flavonoids is clear, but the specific microbial pathways of their anti-atherosclerotic effects have not been fully clarified, and stricter experimental verification is needed (35).

### 3.2 Alkaloids

Alkaloids are a diverse class of naturally occurring nitrogen-containing compounds, many of which contain heterocyclic nitrogen atoms and are widely distributed in medicinal plants (36, 37). Compared with that of polyphenol compounds, the gut microbiota-related evidence for alkaloids is based on fewer representative compounds, but some of them have a more concentrated role in atherosclerosis-related microbial metabolism (38).

Berberine is the most prominent example. Due to the poor bioavailability of oral administration, it is considered to act partially on the intestinal cavity and mucosal interface, rather than taking effect through systemic absorption (39). This characteristic makes its anti-atherosclerotic activity highly related to the regulation of gut microbiota (40). Existing studies show that Berberine can inhibit TMA-producing bacteria and reduce TMAO generation from the source (41). At the same time, it enriches SCFA-producing bacteria and partially corrects the imbalance of gut microbiota (42). These microbial changes are often accompanied by a decrease in inflammatory markers, improved lipid metabolism and reduced atherosclerotic burden in animal models, and clinical observations also provide preliminary support (43).

Palmatine is another representative of alkaloid compounds associated with gut microbiota. As an active ingredient of Huanglian, Palmatine can change the composition of the gut microbiota of mice, reduce the abundance of *Desulfovibrio piger* and the serum level of hippuric acid, reduce the level of atherosclerotic metabolites such as hippuric acid, and slow down the progression of plaques (2). These results suggest that alkaloids do not affect atherosclerosis through a single target, but play a role through the coordinated changes of gut microbiota ecology and microbial metabolite spectrum.

However, such literature needs to be interpreted carefully. Unlike polyphenolic compounds, the relevant evidence for alkaloids is concentrated in a few compounds, especially berberine and several structurally related protoberberine alkaloids, which is difficult to summarize at the category level (44–46). In addition, alkaloids often have direct pharmacological effects on host lipid metabolism, inflammation and vascular signals, which may coexist with gut microbiota remodeling rather than be completely dependent on it (47). In general, alkaloids have great potential in regulating the TMA/TMAO pathway, but more complete more complete comparative and translational evidence is needed to confirm their gut microbiota-mediated effects.

### 3.3 Natural fatty acids

Natural fatty acids are an important category of bioactive compounds in the gut microbiota-atherosclerosis axis. Among them, omega-3 polyunsaturated fatty acids (PUFAs), especially eicosapentaenoic acid (EPA) and docosahexaenoic acid (DHA), are the most well-known, which have clear anti-inflammatory, lipid-lowering and endothelial protective effects (48, 49). More and more evidence shows that their cardiovascular benefits also involve the regulation of gut microbiota and its metabolic outputs (50).

Dietary supplementation of fish oil or other omega-3-rich preparations is associated with an increase in the abundance of relevant bacterial communities that produce anti-inflammatory metabolites and regulation of microbial metabolites such as LPS, bile acids and TMAO-related pathways. These changes can change the intestinal metabolic environment and indirectly affect vascular inflammation and lipid deposition (51). Omega-3 PUFAs can also improve intestinal barrier function by increasing mucosa-associated commensal bacteria, reduce lipopolysaccharide (LPS) translocation into the bloodstream, and reduce systemic low-grade inflammation (52). Animal studies show that fish oil supplementation can improve gut microbial dysbiosis induced by a high-fat diet, optimize lipid profile and reduce the area of atherosclerotic plaques. In addition to long-chain PUFAs, microbial short-chain fatty acids, especially butyrate, are also closely related to this axis. SCFAs such as butyric acid are mainly produced by microbial fermentation, and can also be increased by direct supplementation or prebiotic intervention. They exert anti-inflammatory and anti-atherosclerotic effects and participate in the regulation of atherosclerosis through fatty acid-related pathways (53).

However, there are special interpretation challenges in this category. Natural fatty acids (especially omega-3 PUFAs) have significant and direct effects on host lipid metabolism, inflammation, membrane biology and endothelial function, without necessarily relying on gut microbiota (48, 54, 55). Therefore, compared with poorly absorbed phytochemicals, their gut microbiota-specific mediated effects are more difficult to separate. Although fatty acid interventions clearly affect the gut–vascular axis, the existing evidence supports a mixed model of coexistence of microbial and host-targeting mechanisms, and the relative contributions of the two are still unclear (56).

### 3.4 Other categories

In addition to polyphenols, alkaloids and fatty acids, other categories of natural products such as polysaccharides, saponins and terpenes also show the potential to affect atherosclerosis by regulating gut microbiota (57, 58). The mechanistic significance of these categories is prominent, but their characterization is still not comprehensive (59).

Polysaccharides from plants or fungi are particularly important. As they are often difficult to digest by host enzymes, they act as microbial fermentation substrates and become reasonable regulators of the composition and function of gut microbiota (60). For example, Dendrobium huoshanense polysaccharides can improve the gut microbiota profile of atherosclerosis mouse models, accompanied by enhanced bile acid metabolism and reduced oxidative stress (61). These findings suggest that polysaccharides may affect atherosclerosis mainly by changing the intestinal metabolic environment rather than direct systemic effects (62). Saponins are also closely related to gut microbiota. For example, ginsenoside Rb1 usually needs to be hydrolyzed by microorganisms to produce secondary saponins or glycosides to exert stronger *in vivo* activity. Their lipid reduction and anti-inflammatory effects are closely related to bacteria-dependent biotransformation (63). Similarly, terpenoid quinones such as tanshinones are believed to improve the atherosclerotic phenotype by regulating bacterial composition and inhibiting inflammatory pathways such as NLRP3 inflammasome (64). Although these observations are promising, evidence for this compound class remains immature. Most studies focus on a single compound or a group of small molecules with structural heterogeneity and it is difficult to draw robust categorical conclusions (57).

Therefore, polysaccharides, saponins and terpenoids should now be regarded as emerging categories of gut microbiota-related natural products in atherosclerosis, rather than fully established categories.

### 3.5 Comparative assessment of the evidence

In summary, the existing literature shows that different categories of natural products do not affect atherosclerosis through completely independent gut microbiota-dependent mechanisms. Their effects are repeatedly concentrated in a few common pathways, especially TMA/TMAO metabolic regulation, SCFA production, bile acid homeostasis, intestinal barrier integrity and inflammatory status (20, 35, 65). The main difference between categories lies not in the type of downstream pathways, but in the consistency, mechanistic depth and translational interpretability of the existing evidence (23). Among all categories, polyphenols and flavonoids have the most extensive evidence, but mechanistic overlap and heterogeneity are significant (66). Alkaloids have outstanding prospects in targeting and regulating microbial metabolic pathways especially those related to TMA/TMAO, and the evidence is mainly concentrated in a few representative molecules such as berberine. Natural fatty acids clearly interact with the gut–vascular axis. However, their direct host effects make it more difficult to define the causal relationship of gut microbiota specificity. Other categories such as polysaccharides, saponins and terpenoids provide important mechanistic clues, but they are still relatively preliminary and exploratory (21).

The main limitation in this field is that most studies only infer the participation of gut microbiota through the parallel changes of bacterial community composition, microbial metabolites and atherosclerotic phenotypes, and rarely adopt causal strategies such as antibiotic depletion, germ-free models, fecal microbiota transplantation or targeted verification of specific gut microbiota and enzymes (18). In addition, human research is insufficient, and it is mostly based on surrogate biomarkers, microbial signatures, lipid profiles or inflammatory markers rather than direct vascular endpoints or hard cardiovascular outcomes (20). Therefore, the existing evidence strongly supports the association between natural products, gut microbiota remodeling and the reduction of atherosclerosis-related phenotypes, but the clear causal attribution of specific microbial mechanisms is still an important goal of future research. To avoid treating all natural products as equivalent, **Table 1** summarizes representative interventions according to product class, microbiota-related mechanisms, AS-related outcomes, evidence level, translational status, and major limitations. This product-centered mapping highlights that polyphenols and berberine have relatively stronger mechanistic support, whereas polysaccharides, saponins, terpenoids, and complex formulas remain largely exploratory.

### 3.6 Community-level remodeling of the gut microbiota in atherosclerosis

In addition to regulating specific gut microbiota or metabolite-producing microbiota, natural products can also reduce atherosclerosis by reshaping the gut microbiota at the community level. In models of atherosclerosis, gut microbial dysbiosis usually involves broad ecological disturbances rather than isolated changes in one or two microbiota species. These disturbances include reduced α diversity, altered β diversity, shifts in the Firmicutes/Bacteroidetes ratio, depletion of beneficial anaerobic fermenters, expansion of pro-inflammatory taxa, and impaired ecological stability. These changes may disrupt microbial metabolic networks, enhance endotoxin-related inflammatory signaling, weaken intestinal barrier function, and reduce the production of protective metabolites such as SCFAs.

From this perspective, the anti-atherosclerotic effect of natural products should be understood as the repair of a more balanced and functionally coordinated microbial ecosystem. Many natural products, including polyphenol-rich extracts, flavonoids, alkaloids, polysaccharides and fatty acid-related interventions, have been associated with improved microbial diversity, partial normalization of dominant phyla, enrichment of SCFA-producing bacteria, increased abundance of mucosal-supportive taxa such as *Akkermansia* and *Lactobacillus*, and suppression of TMA-producing or pro-inflammatory bacteria (11, 67–69). In several experimental models, these community-level changes are accompanied by reduced lipid accumulation, decreased inflammatory markers, improved barrier function and attenuated plaque formation (70, 71).

However, commonly used ecological indicators should be interpreted with caution. For example, the Firmicutes/Bacteroidetes ratio cannot be used as a standalone marker of microbiota health, because its biological significance may vary depending on diet, host background and disease model (72). A more meaningful interpretation should integrate microbial diversity, ecological network structure and metabolic output. Overall, natural products may act as ecological modulators that support community stability and functional resilience of the gut microbiota, thereby providing a possible explanation for why different classes of natural products are associated with overlapping vascular-related effects despite acting on distinct microbial targets (73, 74).

### 3.7 Conflicting findings, neutral evidence, and methodological sources of heterogeneity

Although the overall literature supports that natural products have a beneficial effect on the relevant regulation of atherosclerotic gut microbiota, the evidence is not completely consistent and cannot be interpreted as one-way confirmation (65, 75). In different studies, the magnitude or even direction of the gut microbiota change is not always the same, and the most beneficial gut microbiota varies significantly between models, diets and interventions (76, 77). In some reports, the improvement of blood lipids, inflammatory markers or plaque load is more significant than the accompanying changes in gut microbiota, suggesting that the partially observed benefits may reflect the direct host-targeted effect, not just the remodeling of the gut microbiota (78). On the contrary, some studies have reported extensive changes in the gut microbiota, but have not confirmed that these changes are related to the mechanism of vascular improvement (22). Therefore, not all good atherosclerosis phenotypes can be regarded as equally strong evidence of the gut microbiota-mediated mechanism.

In addition to regulating single bacterial taxa and beneficial anaerobic bacteria the emerging theme of natural products and gut microbiota interaction has attracted extensive attention. Natural products can not only directly regulate the activity of specific gut microbiota but also affect the metabolic function of the host through the microbial metabolic network thus exerting anti atherosclerotic effects. However the heterogeneity of research methodology further increases the difficulty of interpretation. Existing studies use different analysis platforms including 16S rRNA sequencing shotgun metagenome targeted metabolites untargeted metabolites and a variety of multi omic combinations each providing different levels of gut microbiota and functional resolution (79). Differences in sampling sites sequencing depth DNA extraction protocols amplification variable regions reference databases and bioinformatics analysis processes may also significantly affect the reported gut microbiota profile (80, 81). Biological heterogeneity is equally important including animal background dietary composition choline and carnitine exposure intervention preparations dosage and course differences. In human studies baseline diet medication individual differences and gut microbiota compliance further increase noise which may obscure repeatable signals.

In summary these limitations suggest that if only supportive results are emphasized it is easy to overinterpret this field. A balanced interpretation of the literature shows that natural products are promising regulators of the gut microbiota atherosclerosis axis but the intensity repeatability and causal interpretability of the evidence vary significantly between different compounds and research designs. Future research requires stricter methodological standardization more rigorous diet control more consistent gut microbiota and metabolome profiles and more widespread use of causal methods such as bacterial depletion fecal microbiota transplantation germ-free verification and enzyme level analysis.

## 4 Mechanisms by which natural products alleviate atherosclerosis via the gut microbiota

Natural products exert anti-atherosclerotic effects through multi-target and multi-pathway interactions, and gut microbiota may act as a key intermediary in this process. Gut microbiota-derived metabolites are not separate from inflammatory or immune mechanisms but serve as the upstream link connecting gut microbiota remodeling and downstream host responses (82). Under this framework, natural products first reshape the composition and function of gut microbiota, and then modulate major metabolic pathways including SCFAs, bile acids, TMA/TMAO and tryptophan-derived metabolites. They further regulate intestinal barrier integrity, inflammatory status, immune function, oxidative stress and lipid metabolism during the progression of atherosclerosis.

### 4.1 Microbiota-derived metabolites as upstream mediators of anti-atherosclerotic effects

Gut microbiota-derived metabolites are the main molecular interface between gut microbiota remodeling and host vascular phenotype in atherosclerosis. In general, atherosclerosis is associated with reduced levels of protective metabolites such as SCFAs and some bile acid derivatives, and increased accumulation of harmful products such as TMAO. Natural products can shift this metabolic balance toward a low-inflammatory and metabolically favorable state by regulating microbial substrate utilization, enzymatic activity and functional gut microbiota abundance (**Table 2**).

**Table 2:** Natural products targeting gut microbiota–mediated pathways in atherosclerosis.

Mechanistic modules	Natural product	Regulated microbiota	Molecular target / axis	Cardiovascular outcome	DOI
TMAO Axis Modulation (Gut microbiota –TMA–FMO3–TMAO pathway)	Liuwei Dihuang Formula (LWDH)	↑ *Bifidobacterium;* ↓ TMA-producing bacteria; ↓ *Escherichia coli (cutC)*	Inhibition of TMA synthesis; enhanced TMA degradation; ↓ TMAO	↓ Plasma TMAO; ↓ plaque burden	10.1016/j.phymed.2025.157318
	Polydatin + Hawthorn flavonoids	*↓ Desulfovibrio;* *↓ Coriobacteriaceae;* *↓ Muribaculum*	Suppression of TMA production; ↓ hepatic FMO3 expression	↓ TMAO; ↓ inflammation; ↓ AS lesions	10.3389/fphar.2025.1515485
	Hickory nut polyphenols	↓ CutC gene–harboring bacteria	Direct inhibition of CutC and FMO3; ↓ TMA formation	Attenuation of HFD-induced AS	10.1016/j.phymed.2024.155349
	Palmatine (PAL)	↓ *Desulfovibrio piger*	Phenylalanine–hippuric acid metabolic axis modulation	↓ inflammatory cytokines; ↓ plaque area	10.1016/j.phrs.2024.107413
SCFA Production & Gut Barrier Axis (Gut–vessel axis)	Dietary fiber (Pectin)	*↑ Akkermansia;* *↓ Lactococcus lactis*	↑ Acetate production; inhibition of plaque-resident bacteria	Plaque progression inhibition	10.1016/j.phymed.2025.157373
	Naoxintong capsule (NXT)	↑ Faecalibacterium; ↑ SCFA-related bacteria	↑ SCFA production; intestinal barrier restoration	↓ systemic inflammation; ↓ early AS	10.1016/j.phymed.2024.155662
	Edgeworthia gardneri extract	*↑ Lactobacillus;* *↑ Akkermansia;* *↑ Faecalibacterium*	Gut barrier integrity enhancement; ↓ LPS translocation	↓ lesion size; improved endothelial function	10.1016/j.atherosclerosis.2025.119132
	Houttuynia cordata extract	*↓ Firmicutes;* *↑ Bacteroidetes*	Enrichment of SCFA-producing bacteria	↓ cholesterol; ↓ atherosclerotic plaques	10.3390/nu16193290
Bile Acid / FXR / Metabolic	Tangzhiqing (TZQ)	*↓ Desulfovibrio;* *↑ Lactobacillus;* *↑ Bacteroides*	Bile acid metabolism regulation; FXR–CYP7A1 signaling activation	↓ diabetic atherosclerosis	10.1016/j.jep.2026.121290
	Dendrobium huoshanense polysaccharides (DHP)	*↑ Bifidobacterium;* *↑ Mucispirillum;* *↓ Desulfovibrionaceae*	Bile acid remodeling; Nrf2/HO-1 activation	Plaque stabilization; ↓ oxidative stress	10.1016/j.phymed.2025.156964
Modulation of microbial tryptophan metabolism	Aucubin	↑ *Lactobacillus;* ↑ *Bifidobacterium (indole-producing commensals)*	Shifts tryptophan metabolism toward protective AhR ligands (indole-3-acetic acid, IAA); AhR activation; enhanced tight-junction protein expression; intestinal barrier reinforcement; inhibition of endothelial-mesenchymal transition	↓ atherosclerotic plaque progression; restrained vascular inflammation; ↓ pro-inflammatory cytokine production	10.1016/j.phymed.2024.156122
	Resveratrol (RSV) + Flavonoids (quercetin derivatives, isoquercitrin)	Restored gut microbial diversity; ↑ indole-producing commensal bacteria	Enhanced substrate availability for indole-producing bacteria; promoted generation of AhR-activating indole metabolites; intestinal barrier repair; suppression of systemic inflammatory signaling	↓ LPS translocation; dampened systemic inflammation; attenuated atherogenic progression	10.1016/j.biopha.2026.119328
	Selected alkaloids & phenolic acids (food-medicine homologous compounds)	Reprogrammed gut microbiota related to tryptophan metabolism	Microbiota-dependent reprogramming of tryptophan metabolic networks; AhR pathway modulation	↓ LPS translocation; recalibrated immune signaling; attenuated vascular inflammation	10.1016/j.biopha.2026.119328

#### 4.1.1 Short-chain fatty acids

SCFAs (especially acetic acid, propionic acid and butyric acid) are the most characteristic protective metabolites in the gut–vascular axis. They are produced by microbial fermentation of indigestible substrates and participate in energy homeostasis, lipid metabolism, immune regulation and inflammation control (83). In atherosclerosis, elevated SCFA levels are usually associated with improved intestinal barrier function, reduced inflammatory activity and a better metabolic environment.

A variety of natural products have been reported to enhance SCFA production in experimental atherosclerosis. For example, NXT can increase total fecal SCFAs especially acetic acid and enrich butyrate related fecal microbial genera (84). Peanut skin polyphenols and red wine polyphenols can also boost butyric acid producing bacteria such as *Roseburia* and improve the anti-atherosclerotic phenotype (14, 85). Mechanistically, SCFAs help maintain intestinal barrier integrity, inhibit intestinal and systemic inflammatory signals, and regulate host metabolism through receptors such as GPR41 and GPR43. Therefore, the enrichment of SCFA-producing bacteria is a reasonable pathway to link gut microbiota remodeling and vascular protection (86).

#### 4.1.2 Bile acids

Gut microbiota-dependent bile acid metabolism is another important pathway in atherosclerosis. Gut microbiota interact with primary bile acids through enzymes such as bile salt hydrolase (BSH) to promote their conversion into secondary bile acids thus affecting enterohepatic circulation cholesterol homeostasis and inflammatory signals (87, 88). Natural products can regulate this axis by promoting the conversion of gut microbiota-dependent cholesterol into bile acids and enhancing the excretion of bile acids (89).

Resveratrol (RSV) is a typical representative. In the TMAO-related atherosclerotic model resveratrol can increase *Lactobacillus* and *Bifidobacterium* enhance microbial BSH activity promote bile acid deconjugation and fecal excretion inhibit ileal FXR-FGF15 signaling increase hepatic CYP7A1 expression and thus promote the conversion of biliary sterols into bile acids to reduce atherosclerotic load (89). Liuwei Dihuang Wan (LWDH) also has a similar effect. In summary these findings suggest that natural products can remodel gut microbiota through gut microbiota-dependent bile acid metabolism improve cholesterol clearance and weaken bile acid-related inflammatory signals (15).

#### 4.1.3 TMA/TMAO pathway

The TMA/TMAO axis is one of the most established gut microbiota-related mechanisms in atherosclerosis. Elevated circulating TMAO is closely associated with endothelial dysfunction, amplified inflammation, foam-cell formation, plaque progression and adverse cardiovascular endpoints (90). Therefore inhibiting this pathway has become a core therapeutic strategy targeting gut microbiota. Natural products can lower TMAO through multiple approaches including suppressing TMA-producing bacteria, downregulating key microbial genes such as cutC and cntA, modulating hepatic FMO3-mediated TMA oxidation and in certain cases enhancing microbial TMA degradation. Resveratrol, Danshen, Liuwei Dihuang Wan and particularly berberine have been verified in experimental models to reduce circulating TMAO and ameliorate the atherosclerotic phenotype (43). Berberine exhibits low oral bioavailability which makes its gut microbiota-dependent pharmacological effects highly reasonable. Current evidence indicates that berberine suppresses microbial choline conversion into TMA, inhibits TMA-producing bacteria and weakens cutC and cntA associated activities thereby reducing TMAO levels and improving inflammation as well as lipid related indicators.

Direct inhibitors of microbial TMA lyase provide further mechanistic support. The natural product related compound 3,3-dimethyl-1-butanol (DMB) has first confirmed that non-lethal inhibition of microbial TMA production can alleviate diet-induced atherosclerosis (91). More potent compounds such as fluoromethylcholine (FMC) and iodomethylcholine (IMC) further verify that microbial TMA generation serves as a causal upstream drug target. Although these compounds are not natural products, they offer an important mechanistic benchmark for evaluating gut microbiota regulators including resveratrol and other analogous natural products.

#### 4.1.4 Microbial tryptophan metabolism

Microbial tryptophan metabolism is an emerging but under characterized pathway connecting gut microbiota remodeling and atherosclerosis. Gut microbiota convert tryptophan into indole-derived metabolites including indole-3-acetic acid IAA indole-3-propionate IPA and indole-3-formaldehyde I3A to regulate epithelial homeostasis mucosal immunity and systemic inflammation levels (92). The core mechanism involves activating aromatic hydrocarbon receptors AhR promoting IL-22 secretion enhancing the integrity of tight junctions and inhibiting atherosclerosis-related pro-inflammatory signals (93).

Emerging evidence shows that some natural products can regulate this pathway. Aucubin can enrich indole-producing commensal bacteria and increase protective tryptophan-derived metabolites such as IAA thereby activating AhR-related signals enhancing barrier function inhibiting endothelial mesenchymal transformation and ultimately reducing plaque progression and vascular inflammation (94). Resveratrol and some flavonoids are also associated with improved gut microbiota diversity and increased AhR-activating indole metabolites which support intestinal barrier repair and immune homeostasis. However compared with the SCFA and TMA/TMAO pathways the evidence is still limited and mainly correlation-based. More causal studies are needed to clarify the contribution of this axis to the anti-atherosclerotic effects of natural products.

### 4.2 Downstream host responses mediated by microbial metabolites

Although SCFAs, bile acids, TMA/TMAO and tryptophan-derived metabolites are often discussed separately, their biological effects converge on several overlapping host processes that drive the progression of atherosclerosis. Against this background, the beneficial effects of natural products can be further understood through three downstream areas maintaining intestinal barrier integrity and inhibiting inflammation regulating immune cell function and macrophage behavior and integrating oxidative stress and lipid metabolism homeostasis.

#### 4.2.1 Barrier integrity and inflammation

Chronic inflammation is a key driver of atherosclerosis and is strongly influenced by microbial metabolism (95). The core link between gut microbial dysbiosis and vascular inflammation is impairment of intestinal barrier integrity which promotes translocation of gut-derived inflammatory products especially LPS into the bloodstream. Circulating LPS then amplifies endothelial damage leukocyte recruitment and plaque progression. Therefore restoring intestinal barrier function is an important way for natural products to reduce the inflammatory burden of vascular walls (96).

Natural products appear to achieve this goal through synergistic regulation of gut microbiota composition metabolite output and epithelial structure. The enrichment of beneficial bacteria such as *Lactobacillus* and *Akkermansia* combined with increased levels of barrier-supporting metabolites including SCFAs and AhR-activating tryptophan derivatives can enhance tight junction integrity and reduce circulating LPS. These changes are often accompanied by inhibition of the TLR4/NF-κB axis and reduced levels of pro-inflammatory cytokines such as IL-6 and TNF-α (97).

**Figure 1:**
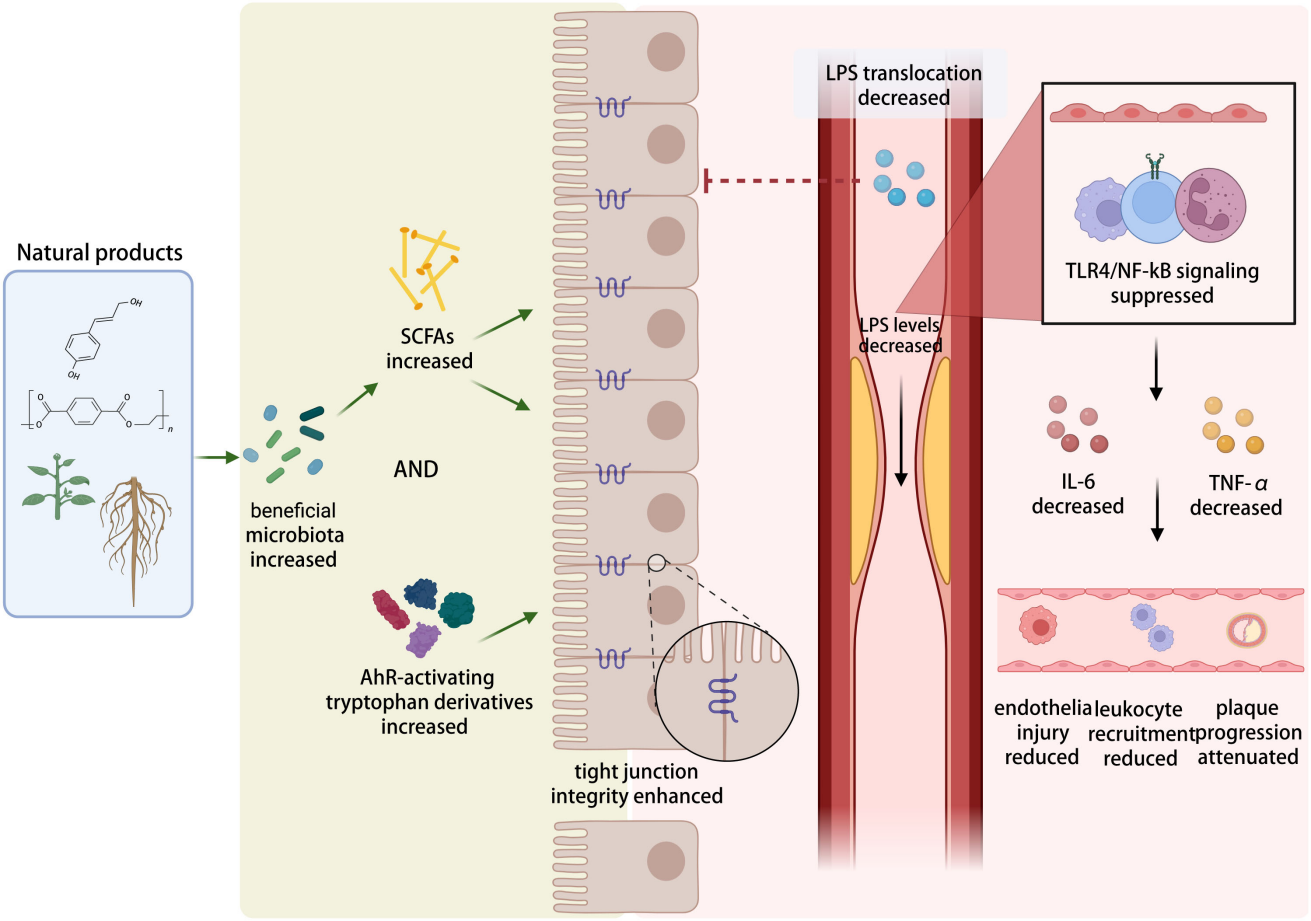
Barrier integrity and inflammation. Natural products alleviate atherosclerosis by remodeling the gut microbiota and regulating microbial metabolism. These effects enhance SCFA production, modulate bile acid and tryptophan pathways, reduce TMA/TMAO formation, improve intestinal barrier integrity, suppress inflammation and oxidative stress, and promote lipid homeostasis, ultimately reducing endothelial injury and plaque progression.

Consistent with this model Edgeworthia extracts may reduce serum LPS levels in atherosclerotic mice and increase the expression of ileal tight junction proteins and these effects disappear after antibiotic depletion. Peanut polyphenols and red wine polyphenols can also reduce inflammatory cytokines while enriching beneficial gut microbiota (97). Traditional prescriptions such as BYHWD and Nei Shuan Mai Soup can also inhibit TLR4/NF-κB-related signaling reduce plaque inflammation and improve gut microbiota and metabolic profiles (16). In summary these findings support that natural products reduce atherosclerosis-related inflammation by restoring intestinal barrier function and reducing gut-derived inflammatory stimulation (17) (**Figure 1**).

#### 4.2.2 Immune regulation and macrophage function

In addition to innate immunity, atherosclerosis involves synergistic alterations in innate and adaptive immunity. Gut microbiota mainly participates in this process by regulating immune cell differentiation, activation thresholds and cytokine production along with microbial metabolites. In particular, SCFAs and microbial tryptophan metabolites help balance anti-inflammatory phenotypes including regulatory T cells and Treg as well as M2 macrophages and pro-inflammatory phenotypes including Th17 cells and M1 macrophages (98).

Natural products can modulate these immune processes by altering the microbial metabolite microenvironment (99). For example, ginseng extracts can increase the proportion of Treg cells in ApoE−/− mice and suppress Th17 responses. This effect may be realized through the gut-microbiota dependent conversion of saponins into active metabolites. Buyang Huanwu Decoction can also lower M1 related markers and elevate the M2 marker Arg-1 which indicates a shift toward plaque stabilizing macrophage phenotypes.

**Figure 2:**
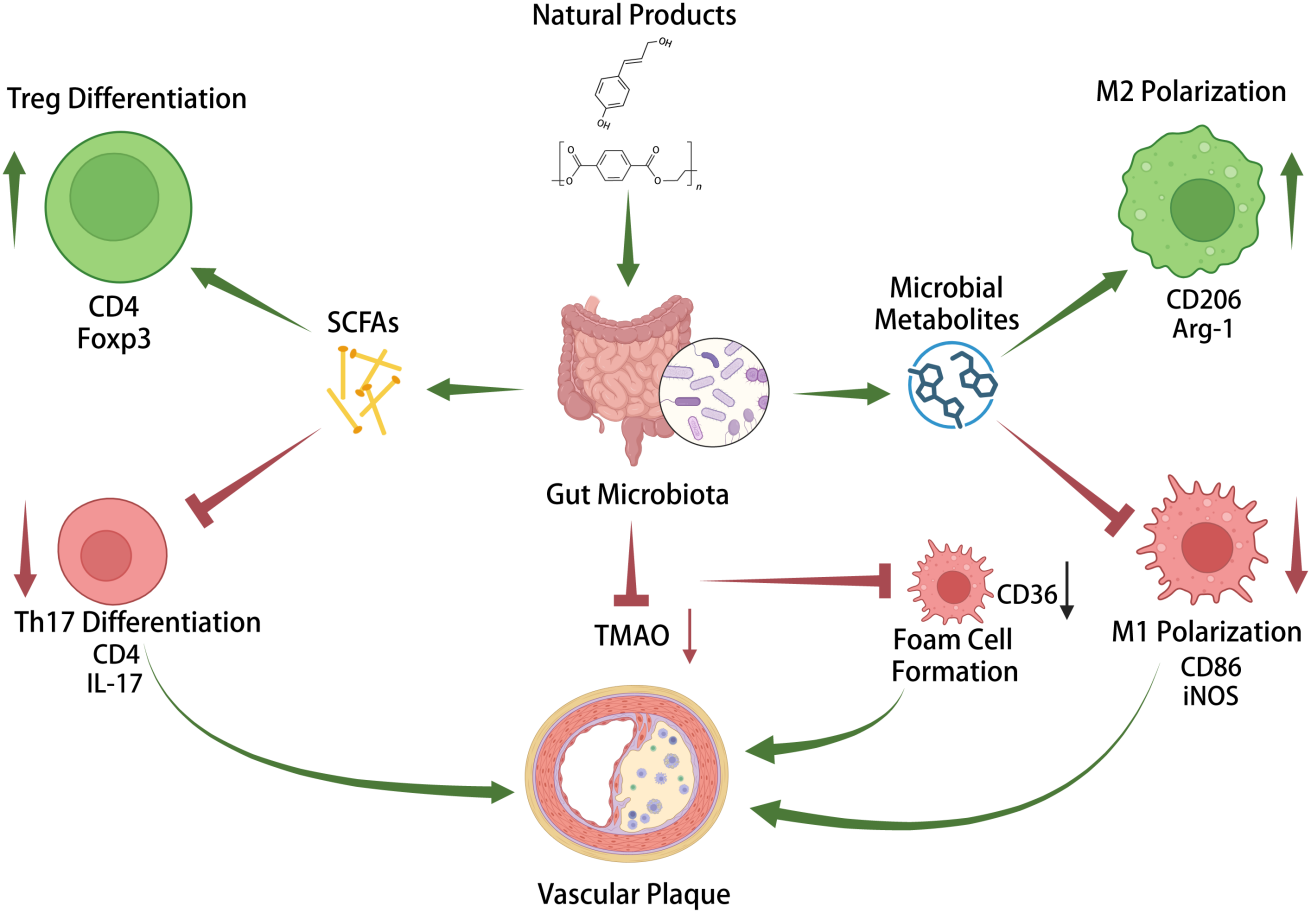
Immune regulation and macrophage function. Natural products modulate gut microbial composition and function, increasing the production of short-chain fatty acids (SCFAs) and other microbial metabolites while reducing trimethylamine N-oxide (TMAO) generation. Elevated SCFAs favor regulatory T-cell (Treg; CD4^+Foxp3^+) differentiation and concomitantly suppress Th17 responses (CD4^+IL-17^+), shifting adaptive immunity toward an anti-inflammatory profile. In parallel, microbial metabolites promote macrophage alternative activation (M2; CD206, Arg-1) and restrain pro-inflammatory polarization (M1; CD86, iNOS). Reduced TMAO signaling further limits foam-cell formation, accompanied by downregulation of CD36-mediated lipid uptake, collectively mitigating plaque progression and contributing to vascular plaque stabilization.

Macrophage regulation is particularly critical because foam cell formation acts as a core pathological event in atherosclerosis. TMAO promotes cholesterol uptake, foam-cell formation and pro-inflammatory macrophage activation. Beneficial metabolites such as SCFAs and specific indole derivatives can restrain these pathological processes. Similarly, resveratrol and tanshinone not only reduce circulating TMAO levels but also downregulate the expression of lipid uptake related proteins such as CD36 and MSR1 (100). These changes restrict foam cell formation and lesion progression. These findings demonstrate that natural products regulate the gut immune axis by mitigating systemic inflammation. They also reshape immune cell differentiation and modulate macrophage behavior through gut-microbiota dependent metabolic signals (64) (**Figure 2**).

#### 4.2.3 Oxidative stress and lipid metabolism

Oxidative stress and lipid metabolism disorders are the main downstream characteristics of atherosclerosis. Both are closely related to gut microbiota-derived metabolites, inflammation and host metabolic signals. Therefore, the antioxidant and lipid-lowering effects of natural products should be understood as part of an integrated gut microbiota-metabolite-host network, rather than an isolated direct effect (101).

Gut microbiota affect cholesterol absorption, bile acid synthesis and transport, host redox signaling, as well as the metabolic pathways of lipid synthesis, oxidation and outflow. Natural products can alleviate oxidative stress, enhance cholesterol clearance, and reduce endothelial damage and lipid peroxidation by improving gut microbiota composition and interrupting metabolic disorders caused by dysregulated metabolic output (102).

**Figure 3:**
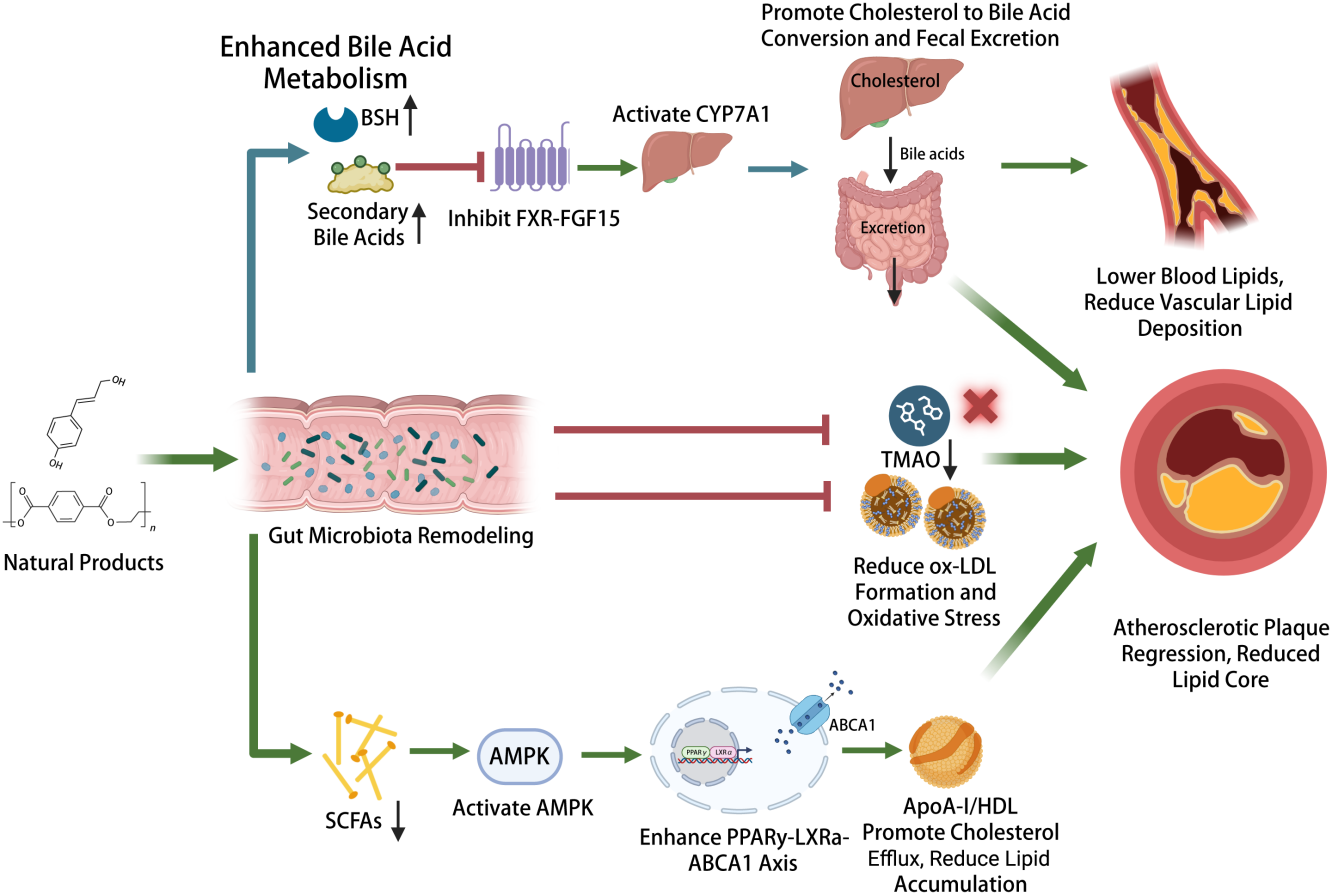
Oxidative stress and lipid metabolism. Natural products remodel the gut microbiota, enhancing bile acid metabolism by increasing bile salt hydrolase (BSH) activity and secondary bile acid production, which suppresses the intestinal FXR–FGF15 axis and promotes hepatic CYP7A1 activation. This coordinated signaling facilitates cholesterol conversion to bile acids and augments fecal bile acid excretion, thereby lowering circulating lipids and limiting vascular lipid deposition. In parallel, microbiota modulation reduces trimethylamine N-oxide (TMAO) and attenuates ox-LDL formation and oxidative stress. Short-chain fatty acids (SCFAs) further activate AMPK and reinforce the PPARγ–LXRα–ABCA1 axis, increasing ApoA-I/HDL–dependent cholesterol efflux and decreasing lipid accumulation within the arterial wall, ultimately contributing to atherosclerotic plaque regression and a reduced lipid core. .

Polyphenolic interventions provide typical cases. Totum-070 may reduce total cholesterol and triglycerides in high-fat diet mice, while reducing intestinal cholesterol absorption, increasing fecal sterol excretion, and improving gut microbiota diversity. Chlorogenic acid can also improve hyperlipidemia and enrich SCFA-producing bacteria, thereby alleviating atherosclerotic lesions (103). At the signal level, a variety of natural products transformed by gut microbiota can activate AMPK, promote fatty acid oxidation, reduce lipid accumulation, and enhance cholesterol outflow. In ApoE−/− mice, red wine polyphenols can increase hepatic AMPK phosphorylation and activate the PPARγ-LXR-ABCA1 axis, which reduces LDL-C, increases HDL-C, and alleviates lipid peroxidation and endothelial damage (8). Hawthorn flavonoids combined with resveratrol can improve insulin resistance and lipid metabolism disorders, thereby reducing atherosclerotic lesions. In general, these findings suggest that natural products can regulate oxidative balance, lipid absorption and cholesterol conversion through gut microbiota remodeling, thereby reducing the substrate load that drives atherosclerosis (104) (**Figure 3**).

### 4.3 Mechanistic convergence and current limitations

In general, the existing evidence supports the convergence model rather than the discrete model (19). Natural products alleviate atherosclerosis by reshaping gut microbiota and altering key metabolic pathways and then coordinately regulating multiple downstream host responses. Under this framework, SCFAs, bile acids, TMA/TMAO and tryptophan-derived metabolites should be regarded as interconnected signal modules that link gut microbiota remodeling with barrier maintenance, inflammatory signaling, immune regulation, oxidative balance and lipid metabolism (77).

**Figure 4:**
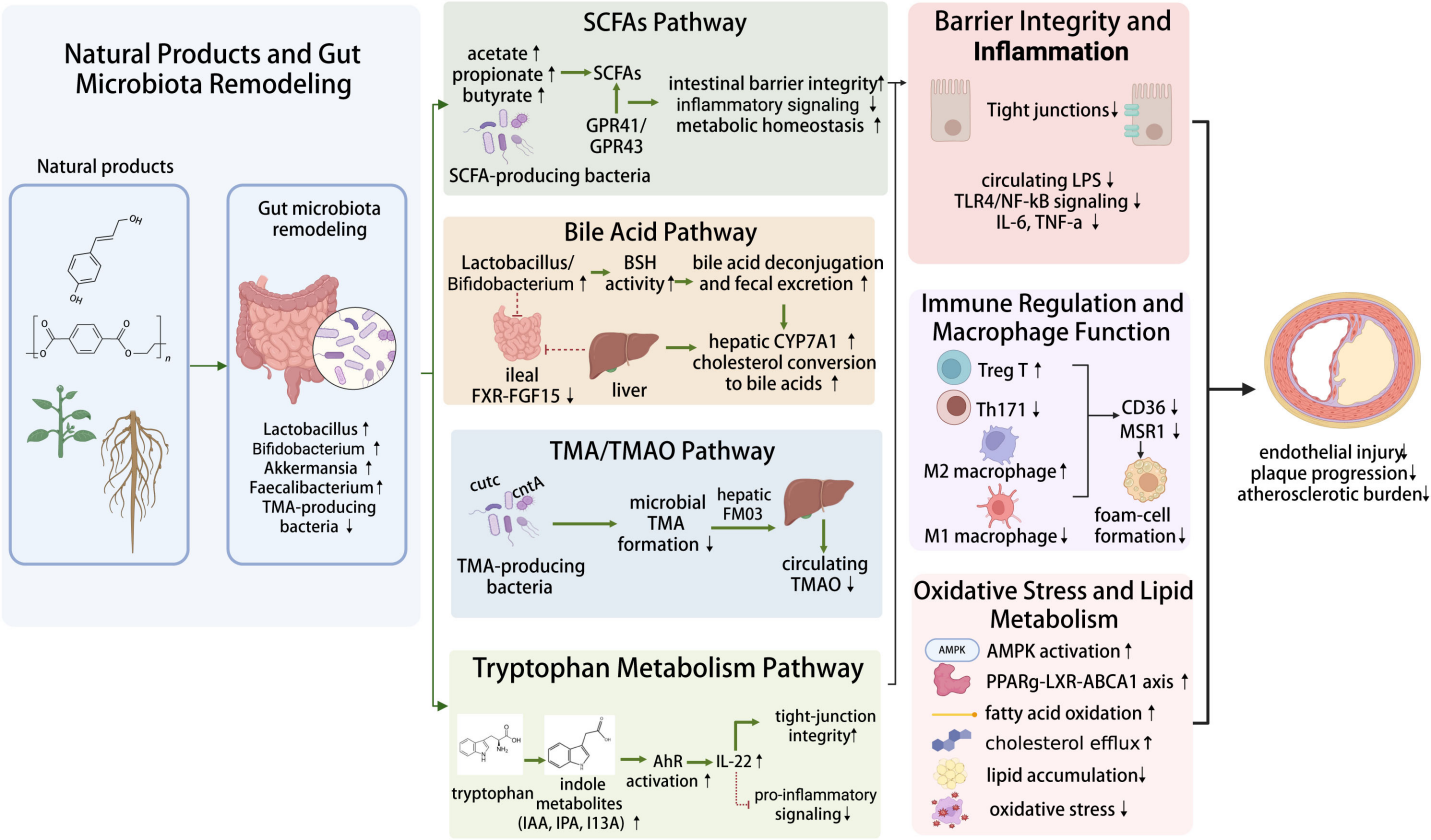
Mechanisms by which natural products alleviate atherosclerosis via the gut microbiota. Natural products remodel the gut microbiota to regulate key metabolic and inflammatory pathways involved in atherosclerosis. By increasing beneficial bacteria and reducing TMA-producing bacteria, they promote SCFA production, modulate bile acid and tryptophan metabolism, reduce TMAO formation, strengthen intestinal barrier integrity, suppress inflammation and oxidative stress, and improve lipid metabolism. These combined effects help reduce endothelial injury, plaque progression, and overall atherosclerotic burden.

However, several limitations still exist. Most available evidence originates from preclinical studies and reliable human data remain limited. Many studies only reveal parallel correlations among gut microbiota composition, metabolite alterations and disease phenotypes and clear causal relationships have not been fully established (75). Moreover, the characterization of gut microbiota tryptophan metabolism under natural product intervention remains less comprehensive than that of SCFA and TMA/TMAO pathways. Future research should emphasize the use of bacterial depletion, fecal microbiota transplantation, germ free models, targeted microbial enzyme analysis and rigorously designed clinical trials for causal verification. This work is crucial to distinguish between the real gut-microbiota dependent mechanism and the broader host targeted effect, and to identify the pathways with the most potential for conversion in the prevention and treatment of atherosclerosis (**Figure 4**).

## 5 Preclinical studies and clinical studies

### 5.1 Preclinical studies

In recent years the research on the natural product gut microbiota atherosclerosis axis has increased significantly accompanied by a large number of animal experiments and cell studies. These studies have provided sufficient preclinical evidence suggesting that natural products may have anti atherosclerotic effects. The research materials include not only single compounds such as resveratrol quercetin quinone and tannin but also compound preparations and complex extracts such as multi flavor Chinese medicinal decoctions and plant preparations (105). The prominent feature of this field is that more attention is paid to confirming the participation of gut microbiota mechanisms rather than just evaluating traditional phenotypic outcomes such as blood lipids and plaque area (73).

To confirm that the gut microbiota is not a spectator the study adopted a variety of experimental strategies including antibiotic mediated gut microbiota depletion germ-free animal models and fecal microbiota transplantation. For example the plaque reduction effect of knotweed extract depends on the integrity of the gut microbiota and its protective effect disappears when the gut microbiota is depleted. In addition when antibiotics are used to deplete the gut microbiota the anti-atherosclerotic effect of knotweed extract is significantly weakened or even disappears. Another example is that the anti-atherosclerotic effect of salvia miltiorrhiza extract is closely related to the gut microbiota and the protective effect is significantly reduced after the gut microbiota is removed. These findings indicate that the anti-atherosclerotic effect of natural products is closely related to the gut microbiota and the participation of the gut microbiota is not a simple bystander role but an indispensable part of the anti-atherosclerotic effect (106).

From a mechanistic perspective, some studies have tracked changes in microbial metabolites including dynamic changes in TMAO and bile acid profiles, while others focus on the reshaping of gut microbiota community composition. 16S rRNA sequencing often reveals an increase in metabolically beneficial bacteria such as *Akkermansia*, *Lactobacillus* and *Roseburia*, along with a reduction in bacteria that produce endotoxins and promote inflammation. A few studies have extended their analysis to the metagenomic and transcriptomic levels, integrating microbial functional genes, host signaling pathways and lesion-related phenotypic characteristics into a more comprehensive mechanistic framework.

In summary the existing preclinical evidence shows a consistent pattern natural products often reshape the gut microbiota ecosystem into a more favorable state accompanied by improved metabolic and inflammatory conditions. In the experimental model these changes are often associated with the occurrence and progression of atherosclerosis. However although these findings support the biological rationality of the gut microbiota-mediated effect their clinical relevance has yet to be confirmed.

### 5.2 Clinical studies

Clinical evidence linking natural products, gut microbiota modulation, and their potential anti-atherosclerotic benefits is emerging. However, most studies have focused on metabolic, inflammatory, or gut microbiota-related surrogate endpoints rather than direct vascular outcomes (107, 108).

For example, a randomized controlled trial of patients with hyperlipidemia showed that NXT combined with statins was more effective than statin monotherapy in improving low-density lipoprotein cholesterol (LDL-C), high-density lipoprotein cholesterol (HDL-C), and triglyceride levels. Combined treatment also reduced the levels of inflammatory markers such as interleukin-6 (IL-6) and tumor necrosis factor-α (TNF-α) by approximately 10–12%. Although the main endpoint of the study focused on lipid metabolism rather than atherosclerotic plaque load, exploratory 16S rRNA sequencing and metabolomic analyses showed that the treatment was associated with specific changes in the gut microbiota profile. Among them, the changes in the abundance of microbiota such as *Streptococcus* and *Veillonella* were significantly associated with the treatment response, suggesting that there was a potential relationship between gut microbiota remodeling and improvement in metabolic and inflammatory status (109, 110).

Similar findings have also been reported for fermented natural products and polyphenol-rich preparations. Fermented Chenpi enzyme preparations may improve lipid profile and increase the abundance of *Bifidobacterium* in feces, thus establishing a link between fermented natural products, microbial community structure, and host metabolic regulation. Other natural compounds such as cyperin have also been shown to regulate the composition of gut microbiota and improve the abundance or functional potential of short-chain fatty acid-producing microbiota, findings that are highly consistent with preclinical model results. In addition, some clinical studies have shown that polyphenolic supplements such as grape seed extract and tea polyphenols may improve endothelial function, accompanied by characteristic changes in gut microbiota. These findings show that the cardiovascular benefits of natural products may not be limited to direct antioxidant or lipid-lowering effects, but may also involve gut microbiota-dependent metabolic and inflammatory signaling pathways (109, 111).

In general, existing clinical studies provide preliminary evidence that natural products may induce beneficial changes in lipid metabolism, inflammatory status, endothelial function. And gut microbiota ecology in people with elevated cardiovascular risk, offering important clues for the clinical translation of preclinical findings.

## 6 Challenges to translation and future research directions

Although preclinical research has provided important evidence for the mechanism of natural products to regulate intestinal flora and reduce atherosclerosis, there are still many challenges in converting these findings into clinical practice. The main obstacles are not only the limited number of clinical studies, but also the complexity of natural product intervention itself, the heterogeneity between individuals in the composition of intestinal flora, and the difficulty of establishing a causal mechanism that depends on intestinal flora in the human body.

### 6.1 Standardization of natural product preparations

One of the major challenges is the standardization of natural product preparations. Unlike single-molecule drugs, most natural products contain a variety of biologically active ingredients. And their composition varies depending on plant origin, harvest season, processing, storage, and extraction methods. These component fluctuations can lead to batch-to-batch variability in efficacy and reduce the repeatability of research results. Therefore, future researches should incorporate strict chemical characterization, quality control, pharmacokinetic evaluation, and dose extra potion or dose exploration strategies. This is especially critical for complex traditional Chinese medicine preparations. Before this kind of preparation is approved for clinical application, its active ingredients, *in vivo* exposure level and long-term safety characteristics must be further clarified (112–117).

### 6.2 Host and microbiota heterogeneity

Host heterogeneity is another key problem. The baseline gut microbiota structure, microbial metabolic ability, diet, lifestyle, comorbidities, and concomitant medications can all affect treatment response. These factors may obscure true therapeutic effects, which is also part of the reason for the inconsistency in gut microbiota characteristics reported across different clinical studies. Therefore, future clinical trials should adopt more precise patient stratification strategies, implement standardized diet and medication control, and carry out paired microbiome-metabolome analyses. Such researches design help identify treatment responders and clarify whether specific benefits depend on baseline microbial characteristics, such as microbial diversity, TMA-producing capacity, and the abundance or functional capacity of short-chain fatty acid-producing taxa (118).

### 6.3 Clinical trial design and endpoint selection

Clinical trials design still needs to be further improved. Most existing human studies mainly take changes in the composition of blood lipid levels, inflammatory biomarkers or intestinal flora as alternative endpoints, while there are few evaluations for direct vascular imaging outcomes and cardiovascular event endpoints. To improve translational value, future randomized controlled trials should have the characteristics of sufficient sample size and longer intervention time, systematically monitor the compliance and safety of the subjects, and incorporate clinically significant vascular imaging outcomes or cardiovascular endpoints.

### 6.4 Combination strategies and safety considerations

At present, natural products are more suitable as an adjunctive interventions for existing cardiovascular treatment regimens rather than alternative therapies. For example, some studies have shown that combining natural products with statins is superior to statin monotherapy in improving metabolic and inflammatory indicators, suggesting its potential value as an add-on strategy. Special attention should be paid to the potential hepatotoxicity and nephrotoxicity of natural products, as well as their interactions with common cardiovascular drugs such as statins, anticoagulants, and proprotein convertase subtilisin/kexin type 9 (PCSK9) inhibitors (119–121). In addition, the strategy of combining natural products with probiotics, prebiotics, synbiotics, engineered bacteria, or microbiota-targeted small molecules may provide new opportunities to reshape the gut microecosystem, enhance intestinal barrier function, and promote the production of beneficial metabolites such as short-chain fatty acids (110, 122).

### 6.5 Multi-omics approaches and causal validation

Beyond combination strategies, the progress of analytical technology and novel therapeutic approaches is opening up new ways for microbiota-targeted interventions. The development of metagenomics, metabolomics, and other multi-omics technologies provides important tools for future mechanistic research. These technologies may link microbial genes, metabolites, and host receptors with downstream vascular responses, promoting the transformation of this field from gut microbiota taxonomic description to functional analysis. Computational biology and artificial intelligence technologies may further help identify key microbial modules, predict treatment responses, and optimize intervention combinations. Meanwhile, emerging strategies such as non-lethal inhibitors of microbial trimethylamine production, small molecules targeting trimethylamine lyase, engineered probiotics, and gut microbiota-based delivery systems are expected to extend insights gained from natural product research to a broader microbiota-targeted therapeutic platform (65, 123–125).

In a word, building a clear causal chain between exposure to natural products, gut microbiota remodeling, microbial metabolite changes and cardiovascular clinical benefits will become the core cornerstone of future translational medical research and development in this field.

## 7 Conclusions

Natural products are potential factors for gut microbiota to regulate atherosclerosis. As summarized in this review, the potential anti-atherosclerotic effects are closely related to the regulation of gut microbiota composition, microbial metabolite generation, intestinal barrier function, lipid metabolism, inflammatory responses, oxidative stress, and immune homeostasis. Natural products do not play a role through a single path, but can play a comprehensive regulatory role in a variety of biological processes involved in the onset of atherosclerosis. These characteristics highlight their important value in elucidating the complex interactions between dietary components, gut microbiota, and host vascular health.

Overall, this review supports the view that modulation of the gut microbiota is an important mechanism through which natural products may contribute to the prevention and intervention of atherosclerosis. Therefore, natural products may provide potential candidates for developing gut microbiota-centered strategies for cardiovascular disease prevention and management, while also offering a theoretical basis for further mechanistic investigations.
